# Effect of heat input on interfacial characterization of the butter joint of hot-rolling CP-Ti/Q235 bimetallic sheets by Laser + CMT

**DOI:** 10.1038/s41598-021-89343-9

**Published:** 2021-05-11

**Authors:** Z. Y. Zhu, Y. L. Liu, G. Q. Gou, W Gao, J. Chen

**Affiliations:** 1 Key Laboratory of Advanced Technologies of Materials, Ministry of Education, Southwest Jiaotong University, Chengdu, 610031 China; 2 Department of Chemical and Materials Engineering, the University of Auckland, PB 92019, Auckland, 1142 New Zealand; 3 Chengdu Industrial and Trade College, Chengdu, 611756 China

**Keywords:** Engineering, Materials science

## Abstract

Composite structures made of 2 mm-thick titanium and 10 mm-thick carbon steel are widely used in infrastructures such as long-distance gas transportation. However, cracking, which is caused by intermetallic compounds (ICs), is a dominate failure mode in welds of this structure. Thus, a common way to improve the in-service life of is reduce the number of ICs. In this paper, we employ a novel hybrid welding method to fabricate composite structures of TA_2_ titanium and Q235 carbon steel. Specifically, Ti and carbon steel is welded by laser and double Cold Metal Transfer (CMT) welding, respectively. The microstructure near the interface of Ti and steel is then examined using SEM, EBSD, EDS, with emphasis on the ICs in terms of chemical elements and morphologies. Results show that FeTi and Fe_2_Ti are the main ICs near the interface, and responsible for the failure of the welds. The effect of welding heat input on the formation of ICs is investigated as well. Results show that ICs are smaller when the heat input is low. Under low heat input circumstance, the tensile strength of the weld can reach up to 420 MPa.

## Introduction

Titanium alloy is one of the good structural materials with low density, high strength, and suitable anti-corrosion properties, however, it lacks the extensive use in the noble material characterization^1^. Carbon steel alloy is a popular structural material with good mechanical properties, weldability, heat stability, and better economics^2^. In some extreme environmental condition like petroleum pipeline transportation and equipment manufacturing^3^, the structures must possess a combination of performances and based on this, the CP-Ti/Q235 bimetallic sheets was made by hot-rolling.

The welding technology is one of the most common methods to connect titanium alloy with steel materials, such as diffusion welding^4–6^, Tungsten inert gas (TIG) welding^7,8^, friction welding^9–13^, soldering^14^, electron beam welding^15–20^, and laser arc welding etc.^21–23^. However, due to large differences in the physical and crystalline chemical properties of the titanium and steel alloys (e.g., specific heat capacity, Ti: 539.1 J/kg·K, Fe: 481.5 J/kg·K; thermal conductivity, Ti:13.8 W/m·K, Fe:66.7 W/MGk; expansion factor, Ti: 8.20 × 10^–6^·K^-1^, Fe:11.76 × 10–6·K^−1^), consequence large distributed residual stress after welding process and would induce cold cracks and delayed cracks or exfoliation of the compound layers. This would generate Fe_2_Ti, FeTi and other brittle alloys compounds and carbides which lead to the difficulty of the welding process^24–31^.

In this field, most of the research has been focused on the role of Titanium alloys with stainless steels. Comparative analysis of the previous literature led us to conclude that Fe_2_Ti_4_O, Fe_2_Ti, FeTi and other brittle alloys compounds and carbides would also be generated. So, the strength of the welded joints is not very strong and decreases when the welding temperature increases. Some scientists have added Cu, Ag, Mg, Mo and their compounds as intermediate transition layers to weld titanium alloys with stainless steels^32–36^. Their results showed that the quantity of brittle compounds is decreased but it cannot be eliminated.

Laser arc or laser-MIG arc welding is the most favorite welding technology which could be used to connect dissimilar metals with its better properties of higher power density, larger ratio of depth-to-width of the welds, and lower welding deformation. Cold metal transmission (CMT) welding technology has lower heat input which avoids drops penetration of the structures and realizes no splash droplet transition and better metallurgical interconnection.

In order to solve the issue of the west to east natural gas transmission, scientists of Pangang Group Research Institute invented new TA2/Q235 bimetallic sheet structures with hot rolling technology. The thickness of the titanium alloy layer has been used as 2 mm along with the 10 mm thickness of the steel alloy layer.

In this work, laser arc welding with CMT welding technology has been optimized with different welding technology tests and employed to systematically investigate the connection of titanium-carbon steel compound structures, the microstructures, distribution of brittle metal alloy compounds, and the thermal strain change process.

## Results and discussion

### Macrostructure and mechanical properties

**Figure 1:**
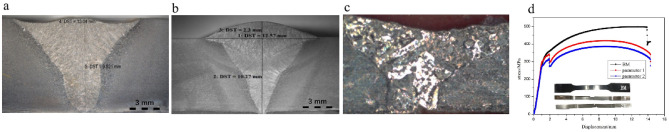
The macrostructure of welded joints. (**a**) Optical image of the sectioned area of the first parameter. (**b**) Optical image of the sectioned area of the second parameter. (**c**) The macrographs of the titanium welded joints. (**d**) The mechanical behavior of joints made of CP-Ti/Q235 bimetallic sheets in different parameters.

The optical macrographs of the sectioned area of the samples are shown in Fig. 1. Figure 1a,b shows the macrographs of the Q235 carbon steel welded joints. The fusion zone was in the perfect forming condition in the first parameter which had lower heat input, weld width: 13.04 mm, penetration: 9.921 mm and no reinforcement. For the second parameter, weld width: 12.57 mm, penetration: 10.27 mm, and reinforcement: 2.3 mm. No any large defect was examined. Figure 1c shows the macrographs of the titanium welded joints. The weld width is about 3.50 mm, the weld width of the back is 1.09 mm, and the bite edge sharpness is 1.09 mm.

Figure 1d shows the results of tensile tests measured for joints processed in different parameters, the ultimate tensile strength (UTS) of the base metal (BM) is slightly higher than that of joints, which is reached 497 MPa. The UTS of the joint in lower heat input (parameter 1) is 420 MPa, which is higher than the UTS of the joint processed in parameter 2(397 MPa). The total elongations of BM and both of joins is 13.2%, 13.8% and 13.9%, respectively.

### Microstructural and phase analysis

**Figure 2:**
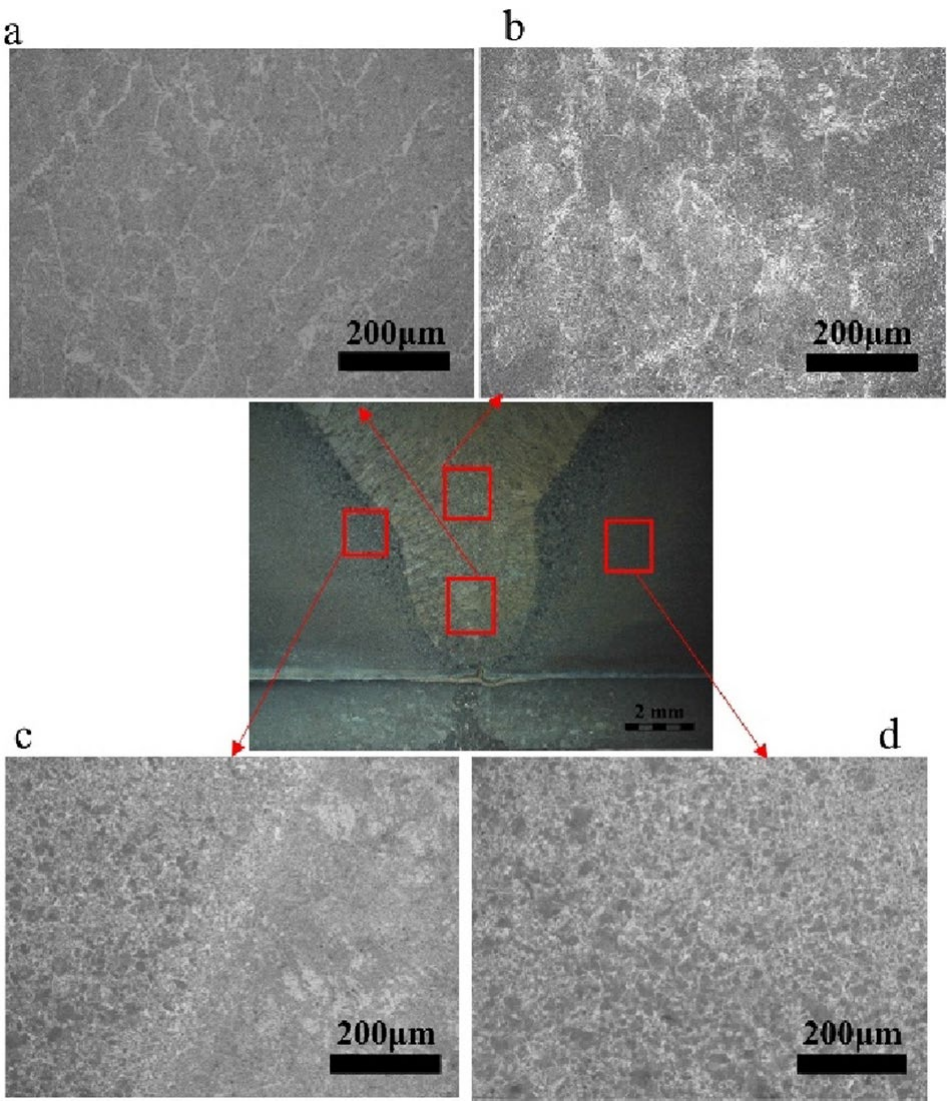
Microstructure of the Q235 steel side for the first welding parameter.

**Figure 3:**
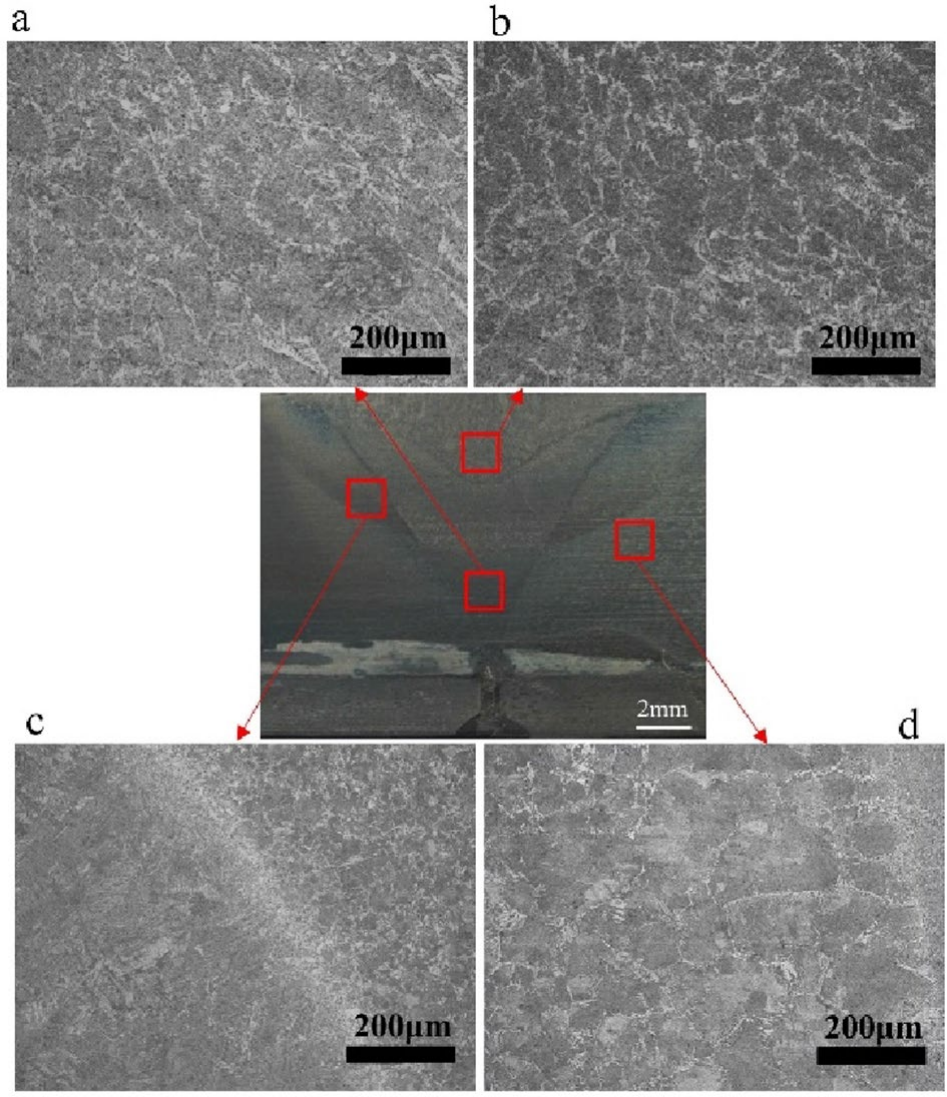
Microstructure of the Q235 steel side for the second welding parameter.

Optical micrographs of the sectioned area on the Q235 side of the steel are shown in Figs. 2 and 3 where the first welding parameter and second welding parameter are shown, respectively.

Ferrites and pearlites are main microstructure for the two welding parameters. The needle-like eutectoid ferrite dissolved along with the austenite grain boundary and the crystal are pearlite in the weld zone, see Figs. 2a,b and 3a,b. The microstructure size of the second layer is much larger than the first layer. For the fusion line microstructures, they are typically distributed just like Widmannstatten structure ferrite with slight decarburization from the center zone. However, the intra-crystalline are acicular ferrite (blue arrow) with pearlite (black arrow) from the left side of the Q235 steel. Widmannstatten structure ferrite develops intra-crystalline growth (red arrow). Moreover, it distributes the proeutectoid ferrite along with grain boundary however, the intra-crystalline are thin ferrite with less Widmannstatten structure ferrite, see Figs. 2c and 3c. The microstructure size of the HAZ zone is much larger than that of the base metals, distributed with less Widmannstatten structure ferrite and thin ferrite.

**Figure 4:**
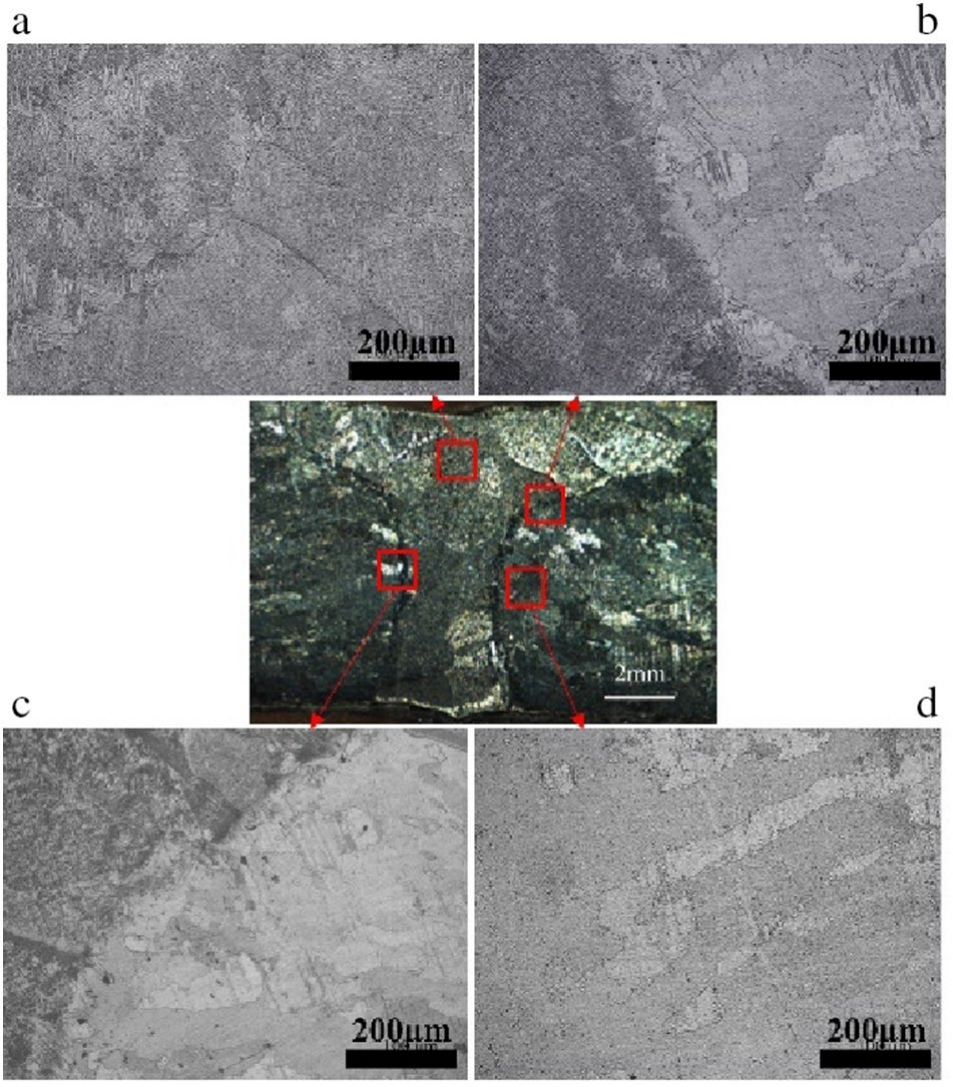
Microstructure of the TA2 side. (**a**) Weld, (**b**) fusion zone, (**c**) fusion zone and (**d**) HAZ zone.

Optical micrographs of the sectioned area on the side of the TA2 are shown in Fig. 4.

The weld (Fig. 4a) is α-Ti with wattle and needle-like microstructure. The bulky columnar crystal of different growth direction and size do not have enough time to expand along the sides, during the solidification and crystallization of the rapid cooling speed of single laser welding. Moreover, this shows the needle-like microstructure. During the high-temperature region, the β-Ti bulky columnar crystal microstructures change to α-Ti martensite and show wattle microstructure.

The fusion zone is composed of battle, needle-like and zigzag α ferrites as shown in Fig. 4b,c. The microstructures undergo phase recrystallization and the grains of the coarse-grained regions (CGR), near the weld region grow rapidly. However, the grains in the fine-grained regions (FGR) which are far away from the weld do not grow efficiently. The HAZ zone is zigzag α ferrites (Fig. 4d) with little larger grain size than that of the base metals and the microstructures changed to martensite, near the fusion line.

**Figure 5:**
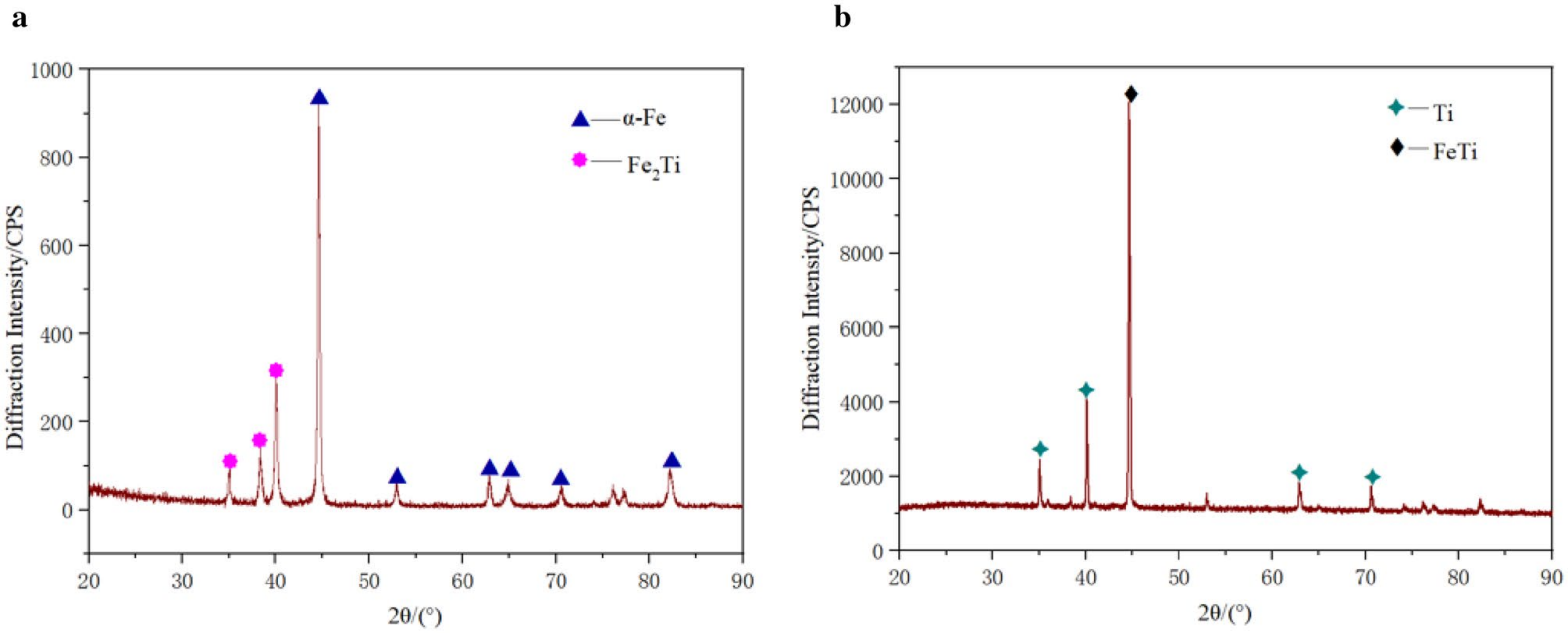
X-ray diffraction pattern of the selected areas in joint: (a) close to the base layer (Q235). (b) Close to the base layer (TA2).

Figure 5 are the results of X-ray diffraction pattern of the selected areas near the bonding surface and close to the base layer(Q235), composite layer (TA2) respectively, which confirms the main occupying element near the base layer of the joint is α-Fe, and near the composite layer is α-Ti, and FeTi and Fe_2_Ti are the main ICs near the interface.

**Figure 6:**
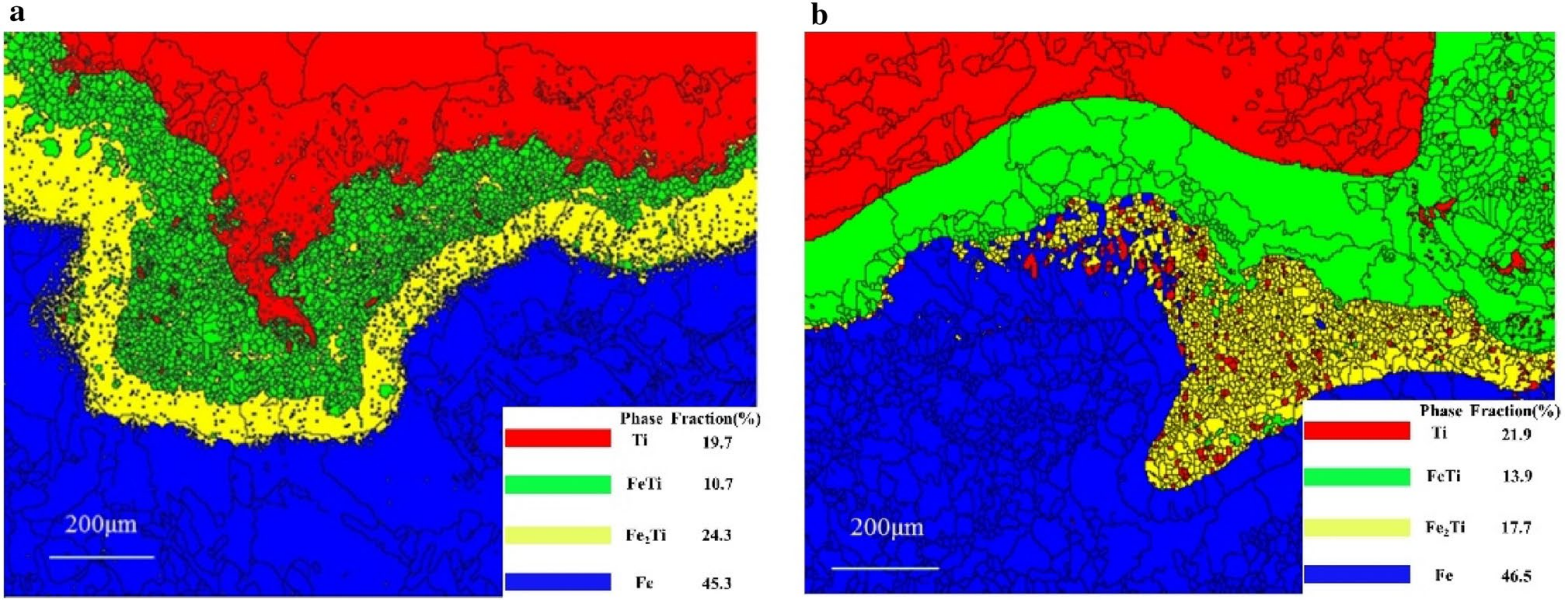
Phase map of the two welding parameters. **(a)** First welding parameter. **(b)** Second welding parameter. Red color: Ti, Green color: FeTi; Yellow color: Fe2Ti; Blue color: Fe.

Phase distribution in the area close to the interface of joints made by the two welding parameters is shown in Fig. 6.

In case of first welding parameter, the Fe phase content is 46.5 wt%, where FeTi is 13.9%, Fe_2_Ti 17.7%, and brittle compounds are 31.6%, where Ti is 21.9%. For the second welding parameter, the Fe phase content is 45.3 wt%, where FeTi is 10.7%, Fe_2_Ti 24.3% and brittle compounds are 35.0% with 24.3% Ti. The high content of brittle compounds is due to increased heat input which promotes the process of diffusion and metallurgy in the interface of the second welding parameter.

**Figure 7:**
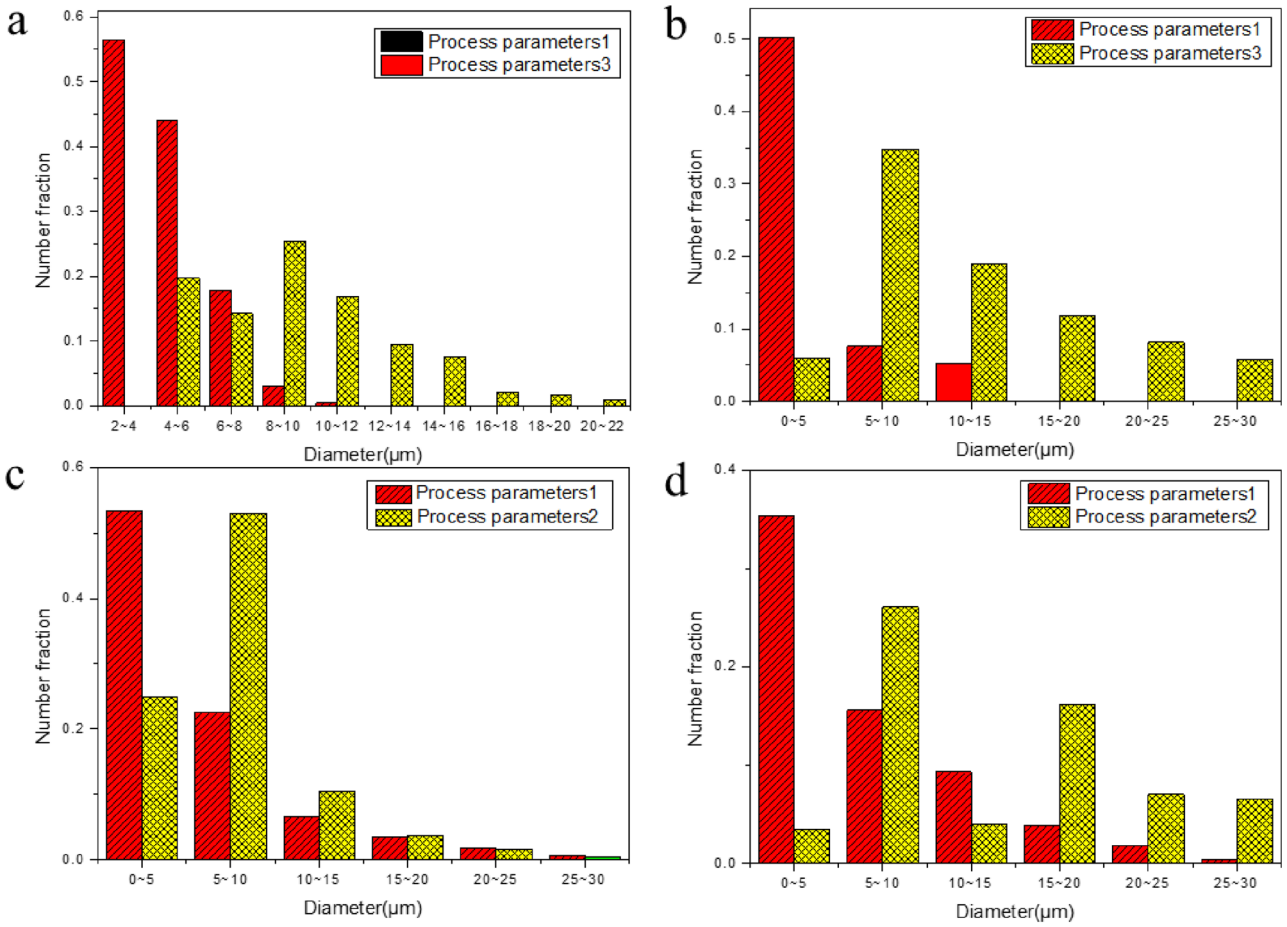
Grain Size of (**a**) Fe_2_Ti, (**b**) FeTi, (**c**) α-Fe, and (**d**) α-Ti grain.

The grain size of the two-welding parameter is shown in Fig. 7.

The mean grain size of α-Fe is 6.5 um in the first welding parameter while it is 19.8 um in the second welding parameter. About 35.4% grains size is distributed from 0 to 5um, 15.6% from 5 to 10 um and some grains size nearly reached to about 30um. For the second welding parameter, the grains size is distributed from 5 to 20um and some grains size reach to 50 um. About 53.4% grains size of Ti is distributed from 0 to 5um, 22.6% grains size is distributed from 5 to 10um and a maximum size of 30um is reached for the first welding parameter. In case of second welding parameter, about 53.1% grains size is distributed from 5 to 10um. The mean grains size of α-Ti for the two welding parameters are 7.2 and 8.5um.

About 50.2% grains size of FeTi is distributed from 0 to 5um and some grains size reached to 15um. For the second welding parameter, about 46.7% grains size is distributed from 5 to 10um while 10 to 30um are a uniform distribution. The mean size of the FeTi of two welding parameters is 2.3 and 15.6um. For the Fe2Ti, the mean size of the two welding parameters is 4.9 and 9.7um. About 96.9% size is distributed from 2 to 6um and some grain size reached to about 12um for the first parameter. However, 76.4% grains size is 6 to 14um and a maximum size of 40um is achieved for the second parameter.

**Figure 8:**
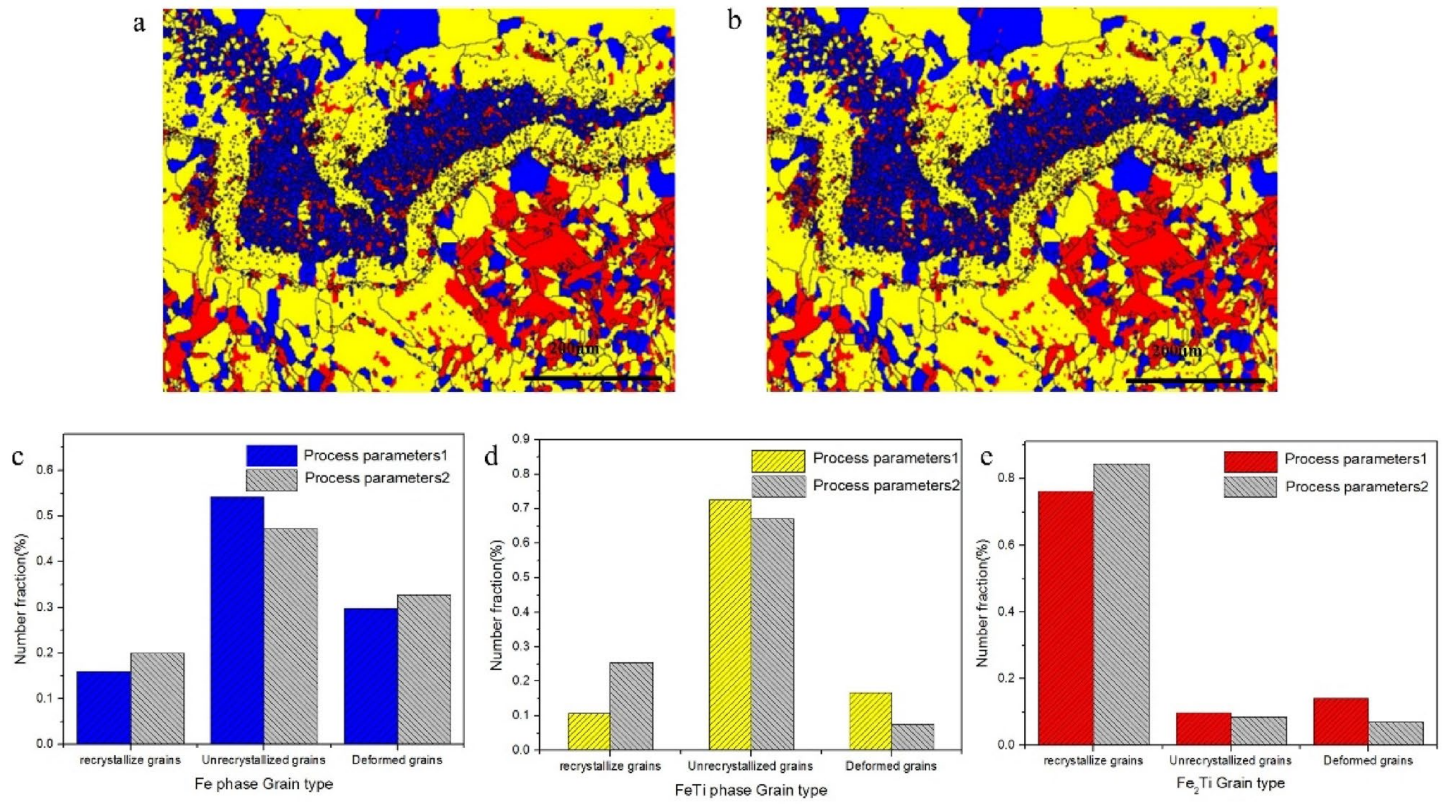
Recrystallization distribution and volume statistics of different phases. **(a)** First welding parameter. **(b)** Second welding parameter, **(c)** Fe recrystallization volume statistics. **(d)** FeTi recrystallization volume statistics. **(e)** Fe_2_Ti recrystallization volume statistics. In this graph, Blue color is for recrystallized grains, Yellow for un-recrystallized grains, and Red for deformed grains.

The recrystallization distribution and volume statistics of different phases are shown in Fig. 8.

For the first and second parameters, the recrystallization volume ratio of Fe phase are 13.3 and 16.0%, for FeTi 10.7 and 25.4%, and for the Fe_2_Ti phase are 76.2 and 84.4%, respectively. This data was calculated with the Channel 5 software. Fe_2_Ti phase distributes with uniform equiaxial grains shape to about full recrystallization. Furthermore, the uniform grains turn into steady flow state at the true stress–strain curves where some extent of deformation exists, which shows the dynamic mode recrystallization mechanism of Fe_2_Ti. on the other hand, in case of Fe and FeTi phases, the low angle boundary (boundary orientation difference ≤ 15°) decreased but high angle boundary (boundary orientation difference ≥ 15°) increased with the increase of heat input. This led us to conclude that the sub-boundary and dislocation density decreased and show static mode recrystallization mechanism of the Fe and FeTi.

**Figure 9:**
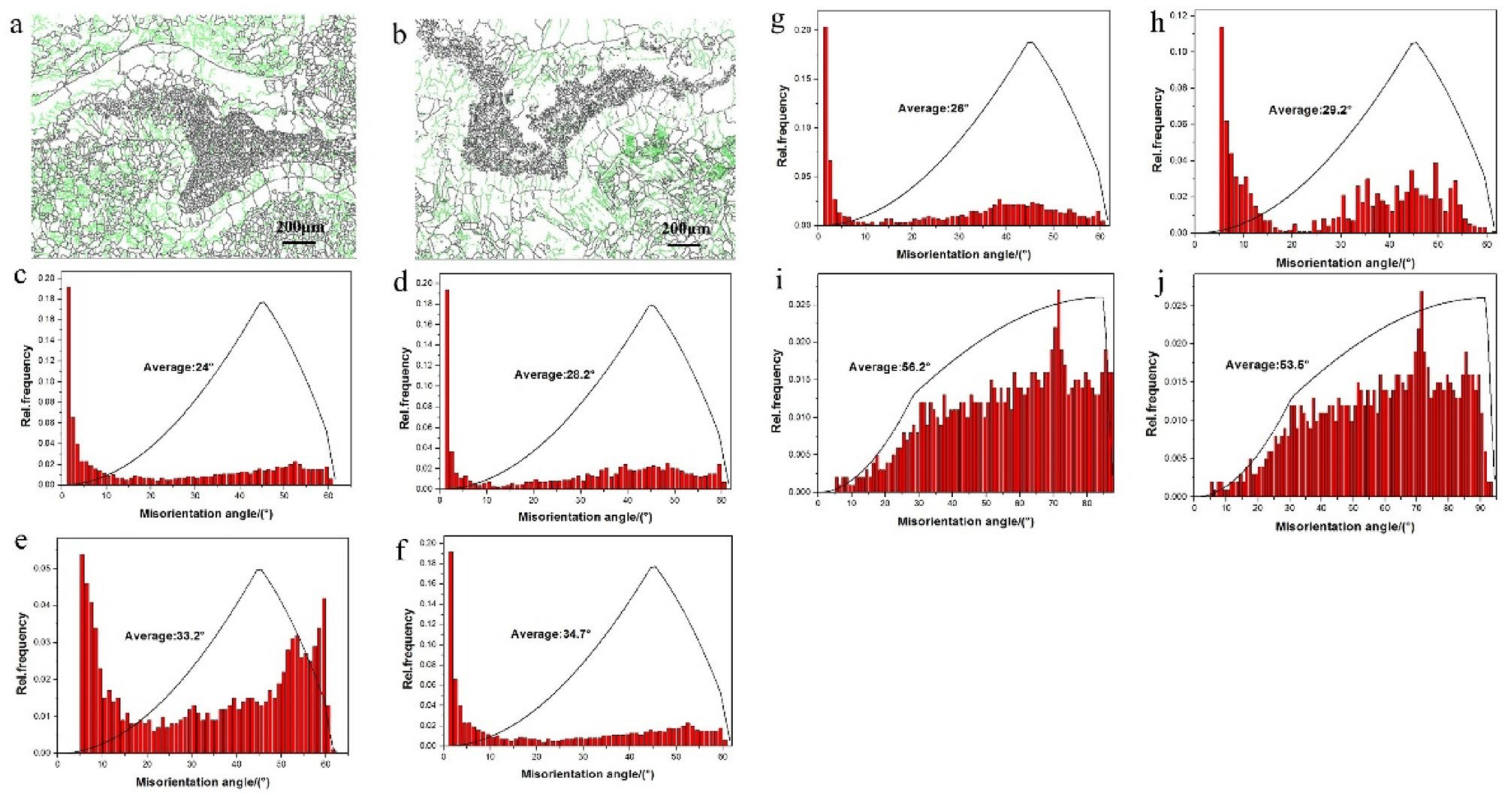
Grains boundary diagrams of different phases. **(a,b)** The grain boundary diagrams of process parameters 1 and 2, respectively. **(c,d)** Fe orientation diagrams of process parameters 1 and 2, respectively. **(e,f)** The Ti orientation diagrams of process parameters 1 and 2, respectively. **(g,h)** The FeTi orientation diagrams of process parameters 1 and 2, respectively, and (**i**,**j**) is the Fe2Ti orientation diagrams of process parameters 1 and 2, respectively.

From the calculation of HKL-Channel software, the Fe phase volume ratio (Fig. 9a–d) at 2° ~ 15° section is 7.4 and 6.9%, separately, while the mean orientation difference is 24 and 33.2%, separately. The volume ratio of Ti phase (Fig. 9a,b,e,f) at 2 ~ 15° section is 5.3 and 3.5%, separately, while the mean orientation difference are 28.2 and 34.7%, separately. In case of the peak value at 60° orientation difference will show crystalline or close-packed hexagonal structure of Ti The volume ratio of FeTi phase (Fig. 9a,b,g,h) at 2 ~ 15° section are 4.2 and 10.3% separately, while the mean orientation difference are 26 and 29.2%, separately. For the Fe_2_Ti phase (Fig. 9a,b,i,j), there exist high angle boundary and the volume ratio at low angle boundary are 0.51 and 0.33%, respectively and the mean orientation difference are 53.5 and 56.2%, respectively. It can be concluded that the change of welding parameters would promote different recrystallization extent which would change low angle boundary to high angle boundary at the sub-grain rotation, responsible for the disappearance of sub-grains. So, the density of dislocation defects decreased with the increase of heat input.

**Figure 10:**
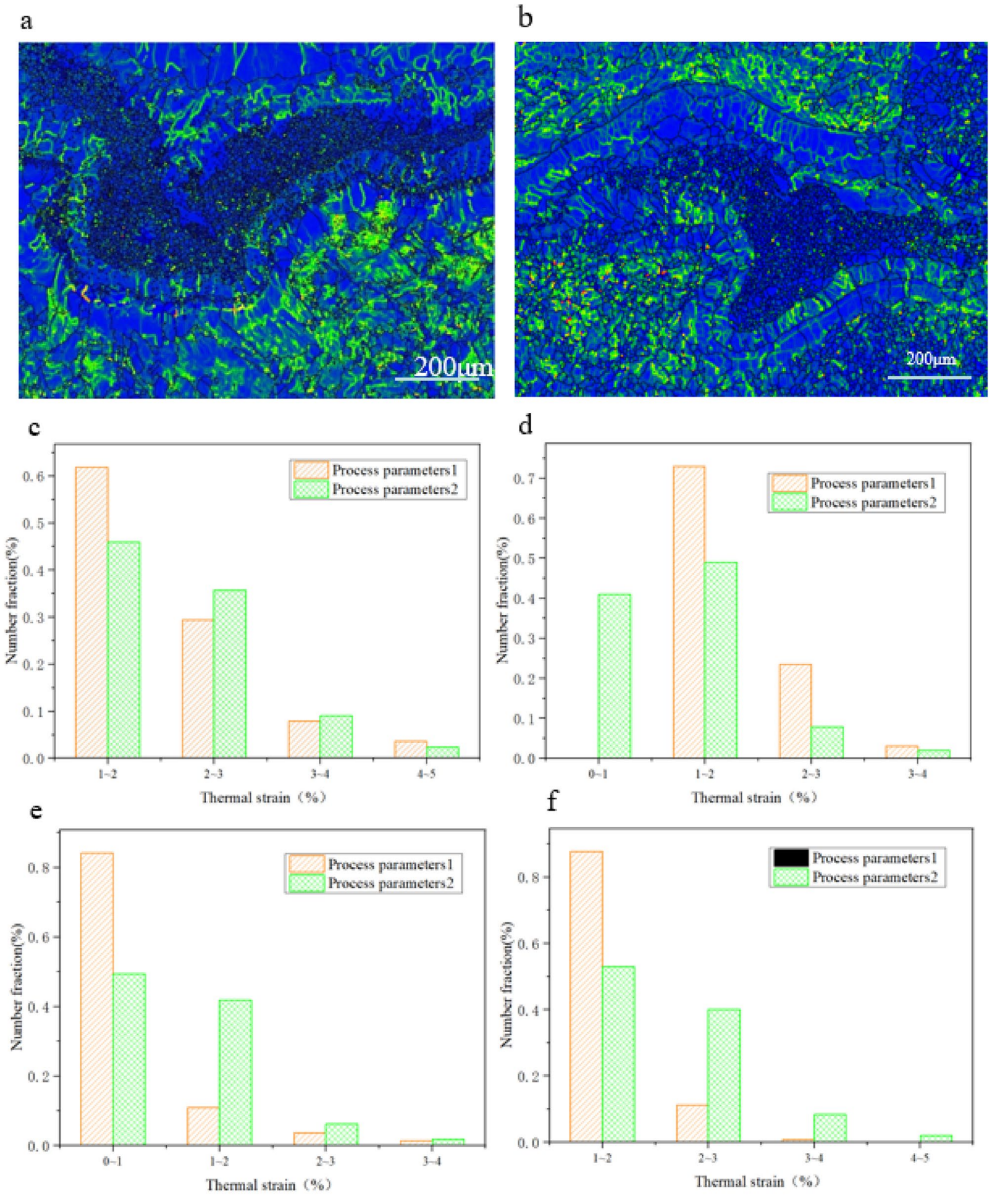
Plastic strain change of different phases: (**a**) Grain boundary diagram of the first parameter (**b**) Grain boundary diagram of the second parameter (**c**) plastic strain of phase Fe (**d**) plastic strain of phase FeTi (**e**) plastic strain of phase Fe_2_Ti (**f**) plastic strain of phase Ti.

The kernel average misorientation (KAM) map in Fig. 10a,b shows that the local plastic strains of the joints in two parameters, the plastics strain is concentrated within the grain boundaries, and presenting an uneven "strip" type distribution. The mean residual strain of Fe phase (Fig. 10c) is 2.12 and 2.25 separately, the mean residual strain of FeTi (Fig. 10d) phase is 1.31 and 1.77 separately, the mean residual strain of Fe_2_Ti (Fig. 10e) is 0.86 and 1.20 separately, the mean residual strain of Ti (Fig. 10f) is 1.48 and 2.25 separately. The residual strain are mainly distributed in the inner of the grains with great inhomogeneity, the grains of larger residual strain interconnected with each other and showed inhomogeneity strip distribution.

## Discussions

**Figure 11:**
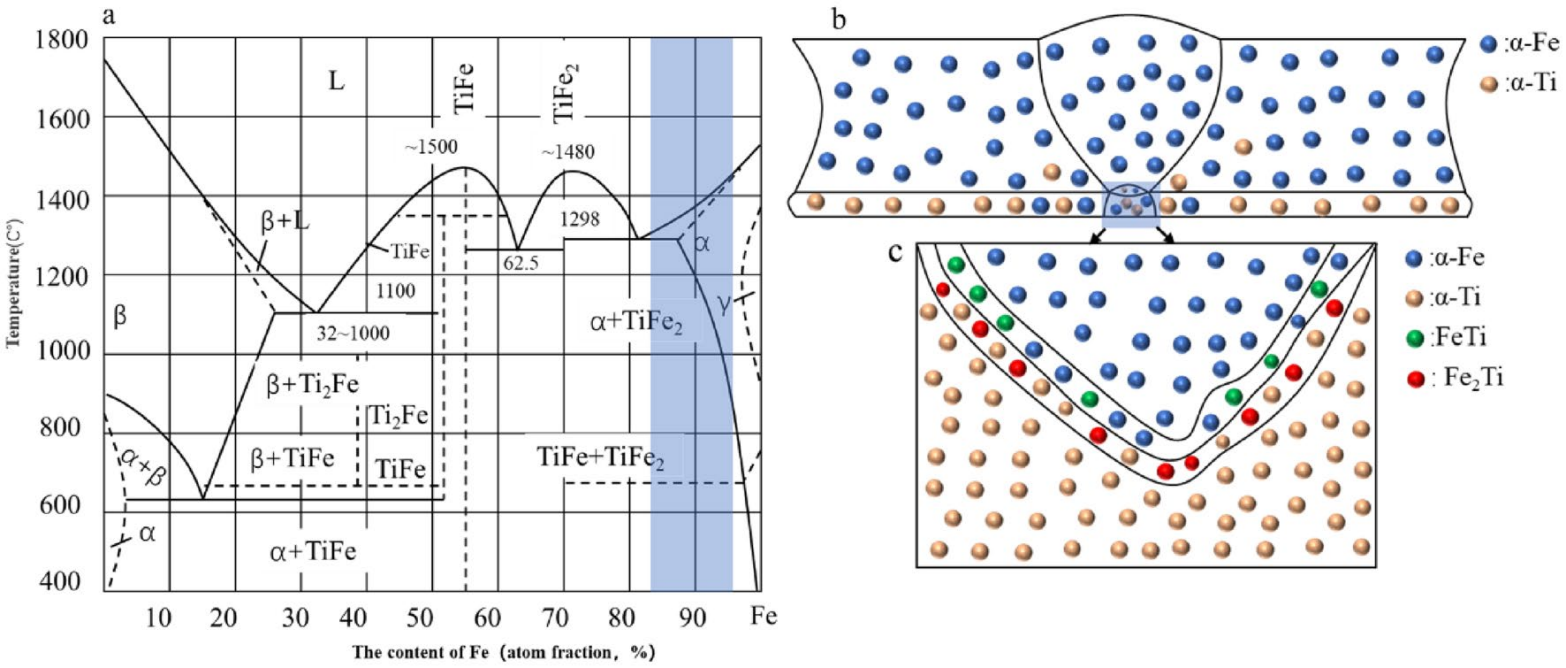
Schematics representation of formation process of FeTi and Fe_2_Ti compounds. **(a)** PHASE diagram of Ti-Fe and their compounds. **(b)** Final elements distribution of Titanium and carbon steel compound welded joints. **(c)** Magnification of (**b**) at the marked zone.

As can be seen from^37^ Fig. 11a, there are some favorable factors which form Ti (for TA2: more than 99wt% titanium and the fusion point is 1677 °C) and Fe (for Q235: more than 99 wt% Fe and the fusion point is 1537 °C) compounds. Both the α-Fe and α-Ti are evenly and non-directionally distributed (Fig. 11b). When the welding process is undergoing, the α-Fe diffuses into TA2 side and the α-Ti diffuses to Q235 side. With the chemical reaction of α-Fe with α-Ti, promoted by the reaction energy, generates high FeTi, Fe_2_Ti (Fig. 11c), and other brittle compounds. As the Fe_2_Ti (Fe ion is Fe^3+^, Ti ion is Ti^6+^) is more stable so, need more energy than that of FeTi (Fe ion is Fe^2+^, Ti ion is Ti^2+^). Finally, the volume ratio of Fe_2_Ti for the second welding parameter is higher than that of the first welding parameter.

## Conclusions

In this work, we have employed laser arc welding and CMT welding technology for the fabrication joints of composite structures made of 2 mm-thick titanium and 10 mm-thick carbon steel. We have investigated the macrostructures, microstructures, Phase distribution, grain morphology and size of the weld interface, recrystallization volume content and grains boundary orientation difference distribution in the sectioned welds, and thermal strain change process through EBSD technology. The following main conclusions are drawn:

1. With the help of appropriate laser arc welding parameter for TA2 and CMT for Q235 carbon steel, the structures of new titanium-carbon steel compounds can be well welded, the joints were fabricated successfully by control the heat input. The UTS of the joint in lower heat input (parameter 1) is 420 MPa, which is higher than the UTS (397 MPa) of the joint processed in parameter 2.
2. FeTi and Fe_2_Ti are the main ICs near the interface in welding zone, the content of ICs is lower and grain size is smaller in the joint processed in parameters with lower heat input, and which is the reasonable for the more excellent mechanical properties.
3. The fraction of recrystallization volume of α-Fe phase, FeTi phase and Fe_2_Ti phase are all added as the increase of heat input. The recrystallization percentage of Fe_2_Ti phase is significantly higher than that of α-Fe phase and FeTi phase, and even nearly complete recrystallization of grains. It suggested that the recrystallization mechanism of Fe_2_Ti phase may be different from that of Fe phase and FeTi phase.
4. Thermal strain reflected directly the residual stresses in the joints, the residual strain is smaller in the joint processed by the parameters with lower heat input, and which is mainly distributed around the grain boundaries of ICs (FeTi and Fe_2_Ti).

## Methods

**Figure 12:**
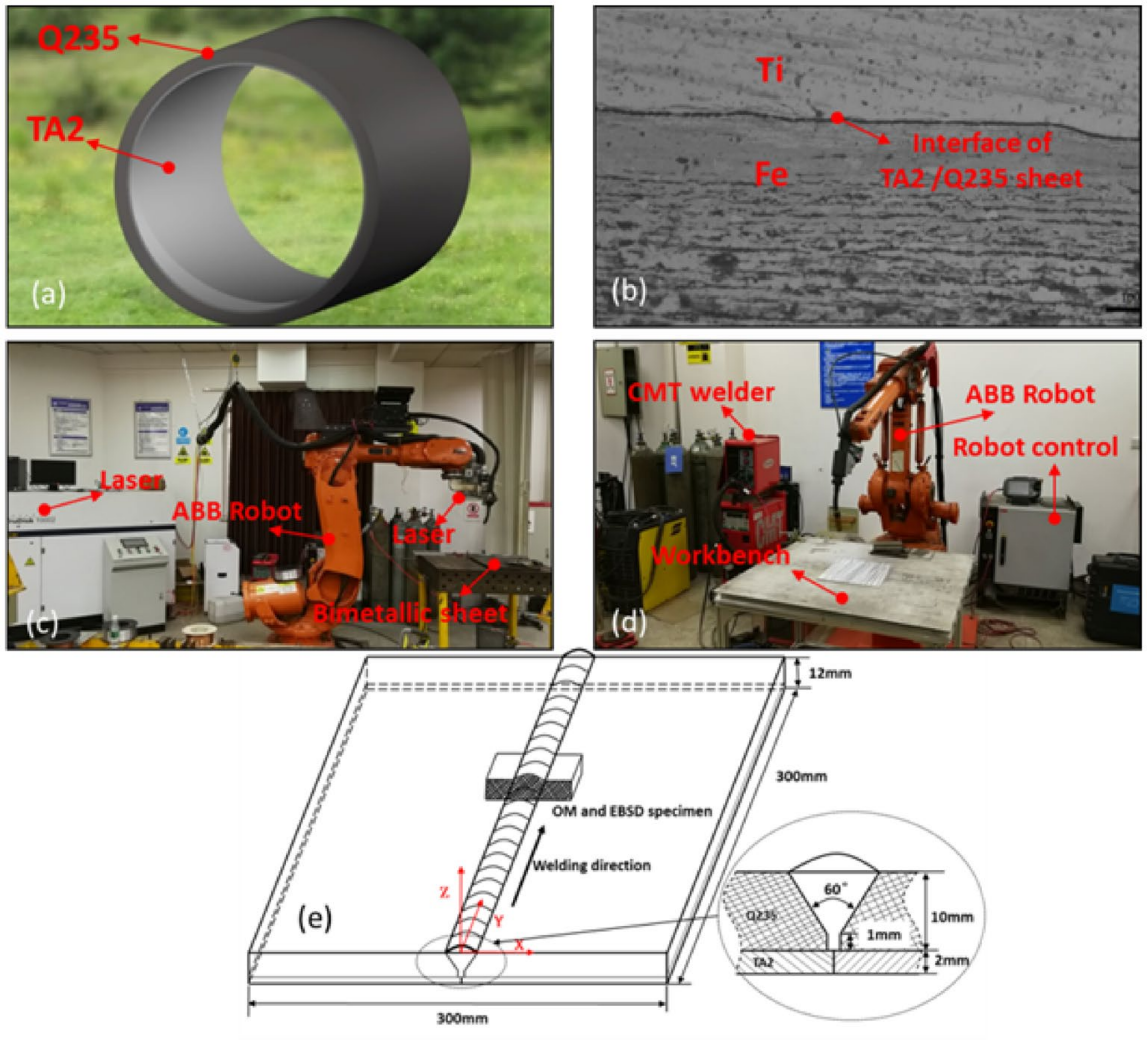
Schematic design for the investigation of titanium alloys with carbon steel compound plates. **(a,b)** From west to east natural gas transmission and the titanium alloys with carbon steel compound plates. **(c)** LAW equipment. **(d)** CMT equipment. **(e)** Schematic of LAHW process and location of sample cutting.

The titanium-steel compound plates are used for the application (Fig. 12a,b) of natural gas transmission, from the west to east, made of single laser arc and CMT process. The carbon steel layer was first made from TPS-4000 CMT welding technology in two layers and the welding wire is ER50-6. After that the titanium layer TA2 was made from a TruDisk 10002 continuous wave disc type laser and a Transpuls Synergic 4000 welding machine (Fig. 12c,d) and 2 KW power was used (welding speed: 30 mm/s, laser angle: 90, gas flow speed:40 L/min). The welding wire is ERTi-2. “V” shaped grooves with 0.5 mm gaps were used with an angle of about 40° ~ 60°. The CMT welding parameters of the carbon steel were divided into two systems. The detailed parameters are listed in Table 1.

**Table 1:** Parameters for CMT welding Q235 carbon steel.

Parameters	Position	Welding current (A)	Welding voltage (V)	Welding speed (mm/s)	Wire feeding speed (mm/s)
Parameters 1	Pass one	265	20	5.0	167
	Pass two	250	20	4.0	150
Parameters 2	Pass one	265	20	3.7	167
	Pass two	250	20	4.0	150

Optical macroscopy, microscopy, phase distribution, grain morphology and size, recrystallization volume content, and grains boundary orientation difference distribution were carried out for microstructure analysis. The samples used for optical microscopy were prepared according to the standard procedure and etched via different reagent. In case of carbon steel, the reagent was 4% volume content nitric acid alcohol while for the titanium, the reagent was 3 mL HF + 10 ml HNO_3_ + 87 mL H_2_O.

Phase distribution, grain morphology and size of the weld interface, recrystallization volume content and grains boundary orientation difference distribution in the sectioned welds were analyzed via electron back-scattered diffraction (EBSD) on an SEM (FEM 6500). The thermal strain change process was also analyzed. The EBSD samples were electrolytically polished in 5% perchloric acid–ethanol solution for 45 s.

The cutting of sample for microstructure analysis was located as shown in Fig. 12e.

## Data Availability

The data that support the findings of this study are available from the corresponding author upon reasonable request.
